# Supplementary material for: A Comparison of Solvent-Based Extraction Methods to Assess the Central Carbon Metabolites in Mouse Bone and Muscle
Source: Metabolites. 2022 May 18;12(5):453. doi: 10.3390/metabo12050453 (PMC9144563; doi:10.3390/metabo12050453)
Supplement: Supplementary file 1 [file metabolites-12-00453-s001.zip › metabolites-1693190-supplementary.pdf]

Supplementary information

Supplementary Table S1: List of metabolite derivatives and their biological group used for reference search. MeOX: Methoxyamine hydrochloride. PPP: Pentose phosphate pathway. SCFA: short chain fatty acid. TCA: Tricarboxylic acid cycle. TMS: Trimethylsilyl derivatives.

Biological group	Metabolite	Detected as
Amino acid	Alanine	2TMS or 3TMS
Amino acid	Asparagine	2TMS
Amino acid	Aspartic acid	2TMS or 3TMS
Amino acid	Cysteine	3TMS
Amino acid	Glycine	2TMS or 3TMS
Amino acid	Isoleucine	1TMS or 2TMS
Amino acid	Leucine	1TMS or 2TMS
Amino acid	Lysine	3TMS
Amino acid	Methionine	1TMS or 2TMS
Amino acid	Phenylalanine	1TMS or 2TMS
Amino acid	Proline	1TMS or 2TMS
Amino acid	Serine	2TMS or 3TMS or 4TMS
Amino acid	Threonine	2TMS or 3TMS
Amino acid	Tryptophan	2TMS
Amino acid	Tyrosine	3TMS
Amino acid	Valine	1TMS
Amino acid	Glutamine	1TMS
Amino acid	Homocysteine	3TMS
Amino acid	Arginine	1TMS
Amino acid	Glucosamine N-acetyl	1MeOx 5TMS
Amino acid derivate	Pyroglutamic acid	2TMS
Glycolysis	Fructose-6-phosphate	1MeOx 6TMS
Glycolysis	Glucose-6-phosphate	1MeOx 6TMS
Glycolysis	Glyceric acid-3-phosphate	4TMS
Glycolysis	Lactic acid	2TMS
Glycolysis	Phosphoenolpyruvic acid	3TMS
Glycolysis	Pyruvic acid	1MeOx 1TMS
Glycolysis	Putrescine	3TMS or 4TMS
TCA	Citric acid	4TMS
TCA	Fumaric acid	2TMS
TCA	2-hydroxy glutaric acid	3TMS
TCA	2-oxo glutaric acid	1MeOx 2TMS
TCA	Malic acid	3TMS
TCA	Succinic acid	2TMS
TCA	Isocitric acid	1MeOx 5TMS
TCA	Aconitic acid	3TMS
Others (Nucleotide)	Adenine	2TMS
Others (Nucleotide)	Uracil	2TMS
Others (Nucleotide)	Uridine	3TMS
Others (Nucleobase)	Adenosine	3TMS or 4TMS
Others (Nucleobase)	Cytosine	2TMS
Others (Nucleobase)	Thymine	2TMS
Others (Glycerol)	Dihydroxyacetone phosphate	1MeOx 3TMS

Others (Glycerol)	Glycerol	3TMS
Others (Glycerol)	Glycerol-3-phosphate	4TMS
Others (Glycerol)	Glyceric acid	3TMS
Others (SCFA)	3-hydroxy butanoic acid	2TMS
Others (SCFA)	4-amino butanoic acid	3TMS
Others (SCFA)	2-amino butanoic acid	2TMS
Others (Sugar alcohol)	Erythritol	4TMS
Others (Sugar alcohol)	Arabitol	5TMS
Others (Alcohol)	Thymine	2TMS
Others (Alcohol)	Sorbitol	1TMS
Others (Carboxylic acid)	Glutaric acid	2TMS
Others (PPP)	Ribose-5-phosphate	1MeOx 5TMS
Others (PPP)	Ribose	1MeOx 4TMS
Others (PPP)	Ribulose-5-phosphate	1MeOx 5TMS
Others (PPP)	Ribitol	5TMS
Others (PPP)	Erythrose-4-phosphate	1MeOx 4TMS
Others (PPP)	Gluconic acid 6-phosphate	7TMS
Others (Urea cycle)	Urea	2TMS
Others (Urea cycle)	Ornithine	3TMS or 4TMS
Others (Sugar)	Trehalose	8TMS
Others (Sugar)	Sucrose	8TMS
Others (Sugar)	Mannose	1MeOx 5TMS
Others (Sugar)	Maltose	1MeOx 8TMS
Others (Sugar)	Glucose	1MeOx 5TMS
Others (Sugar)	Galactitol	6TMS
Others (Sugar)	Acetyl-galactosamine	1MeOx 4TMS
Others (Hydrocarbons)	Xylose	1MeOx 4TMS
Others (Hydrocarbons)	Hypotaurine	3TMS
Others (Vitamin)	Pantothenic acid	3TMS
Others	Maleic Acid	3TMS
Others	Ethanolaminephosphate	4TMS

Supplementary Table S2: List of metabolite's RSD values obtained for each of the protocols performed in bone and muscle tissue.

Metabolite	Bone RSD			Muscle RSD		
	mBD	mBD-low	mMat	mBD	mBD-low	mMat
Alanine	37	18	8	33	42	64
Asparagine	NA	NA	NA	NA	NA	NA
Aspartic acid	25	14	15	28	64	40
Cysteine	17	8	10	25	59	NA
Glycine	30	15	25	18	17	67
Isoleucine	17	NA	NA	60	NA	54
Leucine	32	15	23	45	66	NA
Lysine	43	9	20	58	63	20
Methionine	13	35	26	25	34	52
Phenylalanine	13	50	29	48	48	46
Proline	34	61	51	89	58	53
Serine	25	NA	NA	32	61	56
Threonine	45	44	44	28	37	16

Tryptophan	15	12	10	67	63	39
Tyrosine	26	22	22	63	35	87
Valine	14	27	30	67	40	61
Glutamine	NA	NA	NA	NA	NA	NA
Homocysteine	33	23	52	25	36	26
Arginine	NA	NA	NA	NA	NA	NA
Glucosamine N-acetyl	4	10	21	NA	NA	NA
Pyroglutamic acid	26	15	14	3	11	59
Fructose-6-phosphate	25	33	8	95	69	37
Glucose-6-phosphate	23	20	24	45	60	NA
Glyceric acid-3-phosphate	32	NA	NA	41	86	NA
Lactic acid	11	4	8	65	7	55
Phosphoenolpyruvic acid	9	45	46	29	18	57
Pyruvic acid	18	26	77	29	88	8
Putrescine	50	31	58	45	55	47
Citric acid	4	16	3	32	56	58
Fumaric acid	4	4	6	21	58	25
2-hydroxy glutaric acid	1	13	2	12	15	96
2-oxo glutaric acid	8	NA	NA	36	NA	25
Malic acid	3	4	4	15	19	23
Succinic acid	4	4	5	67	60	70
Isocitric acid	4	17	3	32	55	58
Aconitic acid	NA	NA	NA	NA	NA	NA
Adenine	NA	NA	NA	NA	NA	NA
Uracil	5	27	21	21	58	42
Uridine	10	12	5	31	59	35
Adenosine	37	18	8	28	7	24
Cytosine	31	39	15	27	14	34
Thymine	10	33	15	35	46	NA
Dihydroxyacetone phosphate	30	46	59	36	NA	NA
Glycerol	3	4	4	59	82	21
Glycerol-3-phosphate	9	13	19	55	37	82
Glyceric acid	4	7	2	13	77	27
3-hydroxy butanoic acid	8	18	22	67	20	42
4-amino butanoic acid	NA	NA	NA	NA	NA	NA
2-amino butanoic acid	16	24	NA	86	74	54
Erythritol	NA	NA	NA	NA	NA	NA
Arabitol	NA	NA	NA	NA	NA	NA
Thymine	10	33	15	35	46	NA
Sorbitol	NA	NA	NA	NA	NA	NA
Glutaric acid	35	31	3	20	21	24
Ribose-5-phosphate	33	79	69	56	24	9
Ribose	6	4	3	59	NA	57
Ribulose-5-phosphate	16	10	7	97	75	22
Ribitol	4	18	10	56	83	93
Erythrose-4-phosphate	29	5	1	112	84	NA
Gluconic acid 6-phosphate	13	23	6	NA	NA	NA
Urea	4	11	17	23	10	68
Ornithine	27	60	27	50	69	81
Trehalose	5	64	5	8	23	60
Sucrose	16	23	NA	24	28	52

Mannose	1	4	1	17	53	11
Maltose	4	NA	9	NA	NA	NA
Glucose	2	5	1	NA	NA	NA
Galactitol	3	22	15	13	13	21
Acetyl-galactosamine	4	10	21	NA	NA	NA
Xylose	NA	NA	NA	NA	NA	NA
Hypotaurine	24	29	25	37	18	61
Pantothenic acid	52	57	8	19	16	34
Maleic Acid	3	4	4	18	20	73
Ethanolaminephosphate	24	21	15	51	35	30
